# Discrete Cyclic di-GMP-Dependent Control of Bacterial Predation versus Axenic Growth in *Bdellovibrio bacteriovorus*

**DOI:** 10.1371/journal.ppat.1002493

**Published:** 2012-02-02

**Authors:** Laura Hobley, Rowena K. Y. Fung, Carey Lambert, Maximilian A. T. S. Harris, Jayesh M. Dabhi, Simon S. King, Sarah M. Basford, Kaoru Uchida, Robert Till, Rashidah Ahmad, Shin-Ichi Aizawa, Mark Gomelsky, R. Elizabeth Sockett

**Affiliations:** 1 Centre for Genetics and Genomics, School of Biology, University of Nottingham, Medical School, Nottingham United Kingdom; 2 Department of Molecular Biology, University of Wyoming, Laramie, Wyoming, United States of America; 3 Prefectural University of Hiroshima Department of Life Sciences, Shobara, Hiroshima, Japan; Tufts University School of Medicine, United States of America

## Abstract

*Bdellovibrio bacteriovorus* is a Delta-proteobacterium that oscillates between free-living growth and predation on Gram-negative bacteria including important pathogens of man, animals and plants. After entering the prey periplasm, killing the prey and replicating inside the prey bdelloplast, several motile *B. bacteriovorus* progeny cells emerge. The *B. bacteriovorus* HD100 genome encodes numerous proteins predicted to be involved in signalling via the secondary messenger cyclic di-GMP (c-di-GMP), which is known to affect bacterial lifestyle choices. We investigated the role of c-di-GMP signalling in *B. bacteriovorus*, focussing on the five GGDEF domain proteins that are predicted to function as diguanylyl cyclases initiating c-di-GMP signalling cascades. Inactivation of individual GGDEF domain genes resulted in remarkably distinct phenotypes. Deletion of *dgcB* (*Bd0742*) resulted in a predation impaired, obligately axenic mutant, while deletion of *dgcC (Bd1434)* resulted in the opposite, obligately predatory mutant. Deletion of *dgcA (Bd0367)* abolished gliding motility, producing bacteria capable of predatory invasion but unable to leave the exhausted prey. Complementation was achieved with wild type *dgc* genes, but not with GGAAF versions. Deletion of *cdgA (Bd3125)* substantially slowed predation; this was restored by wild type complementation. Deletion of *dgcD (Bd3766)* had no observable phenotype. *In vitro* assays showed that DgcA, DgcB, and DgcC were diguanylyl cyclases. CdgA lacks enzymatic activity but functions as a c-di-GMP receptor apparently in the DgcB pathway. Activity of DgcD was not detected. Deletion of DgcA strongly decreased the extractable c-di-GMP content of axenic *Bdellovibrio* cells. We show that c-di-GMP signalling pathways are essential for both the free-living and predatory lifestyles of *B. bacteriovorus* and that obligately predatory *dgcC-* can be made lacking a propensity to survive without predation of bacterial pathogens and thus possibly useful in anti-pathogen applications. In contrast to many studies in other bacteria, *Bdellovibrio* shows specificity and lack of overlap in c-di-GMP signalling pathways.

## Author Summary

*Bdellovibrio bacteriovorus* is a tiny bacterium that preys upon other bacteria including pathogenic bacteria that cause infections in humans, animals, or crop plants. *Bdellovibrio* don't attack human, plant or animal cells and so could in future be used as “living antibiotics”. Here we have discovered, using genetics chemical analyses and microscopy, that proteins with a sequence in them called “GGDEF” control whether *Bdellovibrio* grow by preying upon other bacteria or whether they grow “normally” without attacking prey. The GGDEF proteins all synthesise the small signalling molecule cyclic- di GMP, but interestingly the production of this signal has different effects depending on which GGDEF protein makes it. If we remove one GGDEF protein this makes a *Bdellovibrio* that can't eat bacteria anymore and has to survive on environmental nutrients. Removing a different GGDEF protein gives *Bdellovibrio* that can only survive by eating prey bacteria such as pathogens- they lose the ability to eat “normal” nutrients. This is very useful when trying to produce *Bdellovibrio* as a therapy. The correct GGDEF mutant would have to “eat” pathogens only and couldn't grow using the nutrients present in the blood and serum of a wound, for example, so it would be a self-limiting treatment.

## Introduction

Predatory, “attack-phase” cells of *Bdellovibrio bacteriovorus* HD100 use flagellar motility in liquid environments; and gliding motility on solid surfaces, to encounter other Gram-negative bacteria [1], [2]. Prey bacteria include a wide range of pathogens of man, animals and plants, [3], [4] thus *Bdellovibrio* can be seen as “pathogens of pathogens” [5]. *B. bacteriovorus* attach to these prey and enter their periplasms, by mechanisms that remain to be fully understood. Once inside prey, *B. bacteriovorus* become non-motile and degrade prey macromolecules, using them for their own growth and replication (Figure S1 in Text S1). Growth occurs from both poles, giving rise to odd and even numbered progeny by synchronous septation of the elongated multi-nucleoid *B. bacteriovorus* filament, inside the infected, spherical, prey cell which is now called a “bdelloplast” [6]. At the end of predatory growth and septation, *B. bacteriovorus* induce motility once more, and use flagellar motility to emerge from prey in liquid media, or gliding motility to emerge from prey on solid surfaces, and move off, in a non-replicative, “attack phase” to seek more prey encounters. Cultures of *B. bacteriovorus* growing in this predatory or prey/host-dependent (HD) manner require entry to another prey cell to replicate as the “attack-phase” cells have replication suppressed (by as yet unknown control mechanisms) and do not grow using organic nutrients from the external media [7]. Attack phase cells are vibroid and 0.25 µm by 1.25 µm with a single polar sheathed flagellum, they attach to prey at the non-flagellar pole [8].

In the laboratory a small fraction: 1×10^−7^, of attack phase *B. bacteriovorus* populations can also be cultured axenically without prey, as so-called HI (host-independent) cultures on peptone-rich artificial media; here they grow and divide as if inside prey cells (Figure S1 in Text S1) [9]. Natural point mutations in the *Bd0108* Type IVB pilus-like gene product are reported to account for the small fraction of predatory cells that can adopt this “wild type” HI phenotype [10]. Wild-type HI *B. bacteriovorus* HD100 convert readily back to HD cultures, via predatory invasion, if presented with prey cells in the absence of rich media. HI cells of *B. bacteriovorus* are typically longer than the attack phase HD cells, being 2–10 µm long but 0.25–0.3 µm in width; longer cells usually have a serpentine morphology and one or more flagella in polar or other cell sites.

Cyclic di-GMP is a bacterial second messenger that controls various processes. In the Proteobacteria, c-di-GMP controls a lifestyle transition between single, usually motile, cells and surface-attached multicellular communities (biofilms) [11], [12]. It also contributes to the switch between environmental and pathogenic lifestyles in other organisms [13]. C-di-GMP is synthesized by diguanylyl cyclases containing GGDEF domains, and degraded by phosphodiesterases containing EAL or HD-GYP domains. C-di-GMP acts via a variety of receptors (effector proteins) [14]. Among the most common receptors are PilZ domain proteins and a sub-group of GGDEF domain proteins containing so-called I-sites [15]. These sites are present in many diguanylyl cyclases where they function in feedback inhibition. However, a sub-group of the GGDEF domain proteins, which are enzymatically inactive, have evolved that bind c-di-GMP via the I-sites and function as c-di-GMP receptors.

*B. bacteriovorus* HD100 is predicted to have a high c-di-GMP “intelligence” because its 3.8Mb genome contains as many as 15 PilZ domain proteins representing putative c-di-GMP receptors [16], [17], [18]. It is peculiar that the number of predicted diguanylyl cyclases and phosphodiesterases is relatively low, i.e. 5 GGDEF, 1 EAL and 6 HD-GYP domain proteins, which facilitates analysis of c-di-GMP signalling cascades as genes encoding enzymes for c-di-GMP synthesis and degradation can be deleted and the resultant phenotypes tested.

In this study, we carried out a deletion analysis of genes encoding 5 GGDEF domain proteins that may initiate c-di-GMP signalling cascades. Unexpectedly, this analysis produced a discretely different phenotype for each GGDEF domain gene deletion strain. One GGDEF knockout strain was rendered obligately predatory, a phenotype that is very desirable for the future application of *Bdellovibrio* as anti-infective agents to kill pathogenic bacteria [5]. We discovered that individual c-di-GMP signalling pathways control each of the axenic and predatory lifestyles and *B. bacteriovorus* gliding and flagellar motility. The extreme degree of specificity and lack of the overlap among the signalling pathways involving a small diffusible molecule has not been previously observed in bacteria, or believed to be possible. The small (0.25 µm×1.25 µm) *B. bacteriovorus* cell size, which might have been thought to facilitate rapid c-di-GMP equilibration, did not cause nonspecific cross-activation of c-di-GMP cascades. While the mechanisms involving pathway separation are yet to be uncovered, unique cellular localizations of some diguanylyl cyclases and c-di-GMP receptors observed here may contribute to the lack of pathway cross-reactivity. The pathways include one degenerate GGDEF domain protein, with a GVNEF motif, which we found to be a c di-GMP receptor required for the rapid entry of *B. bacteriovorus* into prey cells. Thus c di-GMP signalling has evolved to control the predatory ability of this naturally invasive killer of pathogenic bacteria.

## Results/Discussion

### Bioinformatic analysis of the *B. bacteriovorus* GGDEF domain proteins

We analyzed the sequences of the 5 GGDEF domain proteins encoded in the *B. bacteriovorus* genome using Pfam (Figure S2 in Text S1) The sequences of GGDEF domains of four proteins were very similar to the consensus (Pfam database [19]) and did not contain residues predicted to interfere with the diguanylyl cyclase activity [20]. This suggested that these four proteins are likely enzymatically competent. We designated the *B. bacteriovorus* HD100 locus tags for these proteins [21] as encoding DgcA (Bd0367), DgcB (Bd0742), DgcC (Bd1434) and DgcD (Bd3766), where Dgc stands for diguanylyl cyclase. A genome sequence comparison revealed that DgcA, DgcB and DgcC are conserved between different *Bdellovibrio* strains, whereas gene Bd3766, encoding DgcD, is not conserved in the genome of another *B. bacteriovorus* strain tiberius (Hobley *et al.*, in preparation).

Pfam predicts that [19] DgcA has an N-terminal receiver domain typical of bacterial response regulators (Figure S2 in Text S1), which implies that its activity is regulated by phosphorylation by an unknown histidine kinase. DgcB has an N-terminal forkhead domain, which may be involved in binding of a protein containing a phosphorylated serine or threonine residue [22]. DgcC contains a large N-terminal domain of unknown function. DgcD has a predicted periplasmic N-terminal domain containing 4 tetratricopeptide repeats, TPR domains, flanked by the transmembrane domains.

The GGDEF domain of the fifth protein, Bd3125, was clearly degenerate. Among others, it had two substitutions at key residues in the most conserved GGDEF (Gly-Gly-Asp-Glu-Phe) motif, the catalytic half site required for substrate, GTP, binding, i.e., GGDEF→GVNEF. Thus protein sequence analysis suggested that Bd3125 is not enzymatically competent. Each of the five proteins, including Bd3125, contains an RxxD motif 5 residues upstream of the GGDEF motif, which is a typical sequence and position for the I-site. For the four Dgc proteins, this site likely represents a site for feedback inhibition. Given the likely lack of diguanylyl cyclase activity of Bd3125 but the presence of the I-site, we predicted that it may function as a c-di-GMP receptor (effector) protein, and as such designated it CdgA.

### Biochemical characterization of the *B. bacteriovorus* GGDEF domain proteins

To characterize activities of the GGDEF domain proteins, we first expressed them as fusions to the maltose-binding protein, MBP, in the highly motile strain, *E. coli* MG1655. For DgcD that contains a large transmembrane domain, the fusion was made directly to the GGDEF domain. Ko and co-workers, followed by Ryjenkov and co-workers have shown that elevated c-di-GMP levels inhibit *E. coli* motility in swim agar plates [23], [24]. Each MBP-fusion was tested for the effect it had on *E. coli* motility in swim agar plates. As expected, DgcA, DgcB and DgcC strongly inhibited *E. coli* swimming (Figure S3 in Text S1), consistent with their predicted diguanylyl cyclase activities. However, the MBP-fusion to the GGDEF domain of DgcD did not inhibit swimming, either because its GGDEF domain is enzymatically inactive, or, more likely, because it could not be activated in *E. coli*. Because the *B. bacteriovorus dgcD* knockout produced no observable phenotype, biochemical characterization of DgcD was not pursued. Expression of CdgA produced a significantly larger swim zone than that of the empty vector (Figure S3 in Text S1). Since CdgA cannot possibly act as a c-di-GMP degrading enzyme, we predict that it likely binds c-di-GMP and acts as a c-di-GMP sink, thus effectively decreasing the pool of available c-di-GMP in *E. coli*.

**Figure 1:**
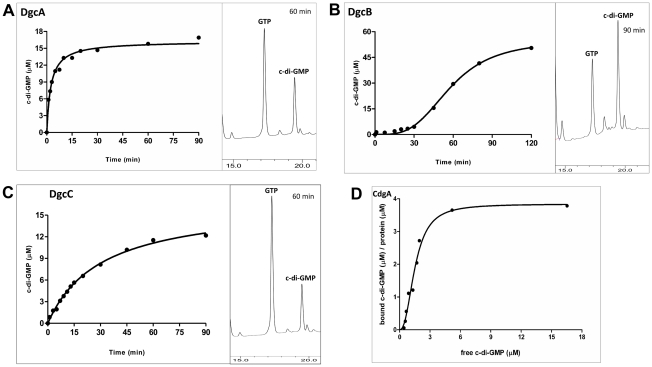
Enzymatic activity of the MBP-fusion proteins. Enzymatic activity of the MBP-fusion to A: DgcA (Bd0367); B: DgcB (Bd0742); C: DgcC (Bd1434); the HPLC profiles show peaks of c-di-GMP (product) and GTP (substrate) at a single time point. D: Saturation plot of equilibrium binding between c-di-GMP and CdgA (Bd3125). Protein-nucleotide binding was examined by equilibrium dialysis in Dispo-Biodialyzer cassettes as described earlier [24]. It appears that CdgA binds 4 molecules of c-di-GMP, which is possible if CdgA were to form tetramers in the presence of c-di-GMP, similar to those described for c-di-GMP-inhibited state of diguanylate cyclases [50].

We purified the MBP-fusions to DgcA, DgcB, DgcC and CdgA and tested diguanylyl cyclase activities and c-di-GMP binding *in vitro*. As expected, DgcA, DgcB and DgcC proved to function as diguanylyl cyclases (Figure 1A–C). Because each of these proteins contains an N-terminal regulatory domain whose activation cannot be ensured in *E. coli*, it is likely that the observed activities of the purified fusions lie between their respective active and inactive states.

Among the three cyclases, DgcB is most sensitive to feedback inhibition by c-di-GMP (Figure 1B). DgcB purified from *E. coli* contained c-di-GMP at approximately 1∶1 protein:c-di-GMP molar ratio. No diguanylyl cyclase activity was observed following addition of the substrate, GTP, to the purified protein. Only after extended (approximately 30 minutes) incubation at 37°C, could the DgcB activity be detected, probably due to unfolding of the c-di-GMP-inhibited DgcB conformation and spontaneous formation of the enzymatically competent DgcB dimer [25].

The CdgA protein containing a degenerate GGDEF domain showed no diguanylyl cyclase activity, which is consistent with the sequence analysis and motility assays in *E. coli* (Figure S3 in Text S1). However, this protein bound c-di-GMP *in vitro* with an apparent K_d_ ∼2 µM (Figure 1D), as measured by equilibrium dialysis [26]. This value is well within the range of intracellular c-di-GMP concentrations observed in bacteria [12]. Therefore, CdgA is a c-di-GMP receptor (effector protein), which, according to the genetic analysis shown below, acts downstream of the diguanylyl cyclase DgcB.

### DgcA signals control the motile escape of *Bdellovibrio* from prey after their digestion, controlling both swarming and swimming motility

**Figure 2:**
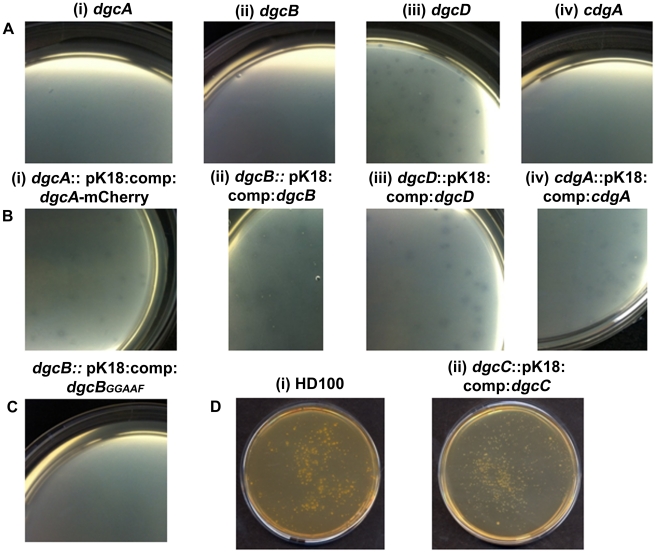
Predatory and axenic growth on agar plates. A: *Bdellovibrio* deletion strains plated onto lawns of prey (i) Δ*dgcA* (ii) Δ*dgcB* (iii) Δ*dgcD* and (iv) Δ*cdgA*. The Δ*dgcD* strain forms plaques in the prey lawns. B: Complementation by single crossover of wild-type and mCherry fusion proteins restores plaquing ability to each deletion mutant: (i) *dgcA*::pK18:comp:*dgcA*-mCherry; (ii) *dgcB*::pK18:comp:*dgcB*; (iii) *dgcD*::pK18:comp:*dgcD*; and (iv) c*dgA*::pK18:comp:c*dgA*. C: Plaquing is not restored to the Δ*dgcB* mutant when a GGAAF mutation is introduced into the complementation plasmid. D: Axenic growth by (i) wild-type *Bdellovibrio*; and (ii) the complemented Δ*dgcC* strain.

An in-frame deletion of *dgcA* was achieved by conjugation of a suicide vector *pK18mobsacB* into *B. bacteriovorus HD100* and subsequent sucrose suicide screening for gene replacement and loss of the plasmid (see materials and methods and Text S1; [2], [27]). The Δ*dgcA* mutant strain could not grow predatorily and did not form plaques (Figure 2Ai) on lawns of prey bacteria, thus it was isolated solely by axenic culturing on peptone-rich media. The Δ*dgcA* mutant was seen by phase contrast microscopy to be non-motile in liquid media.

**Figure 3:**
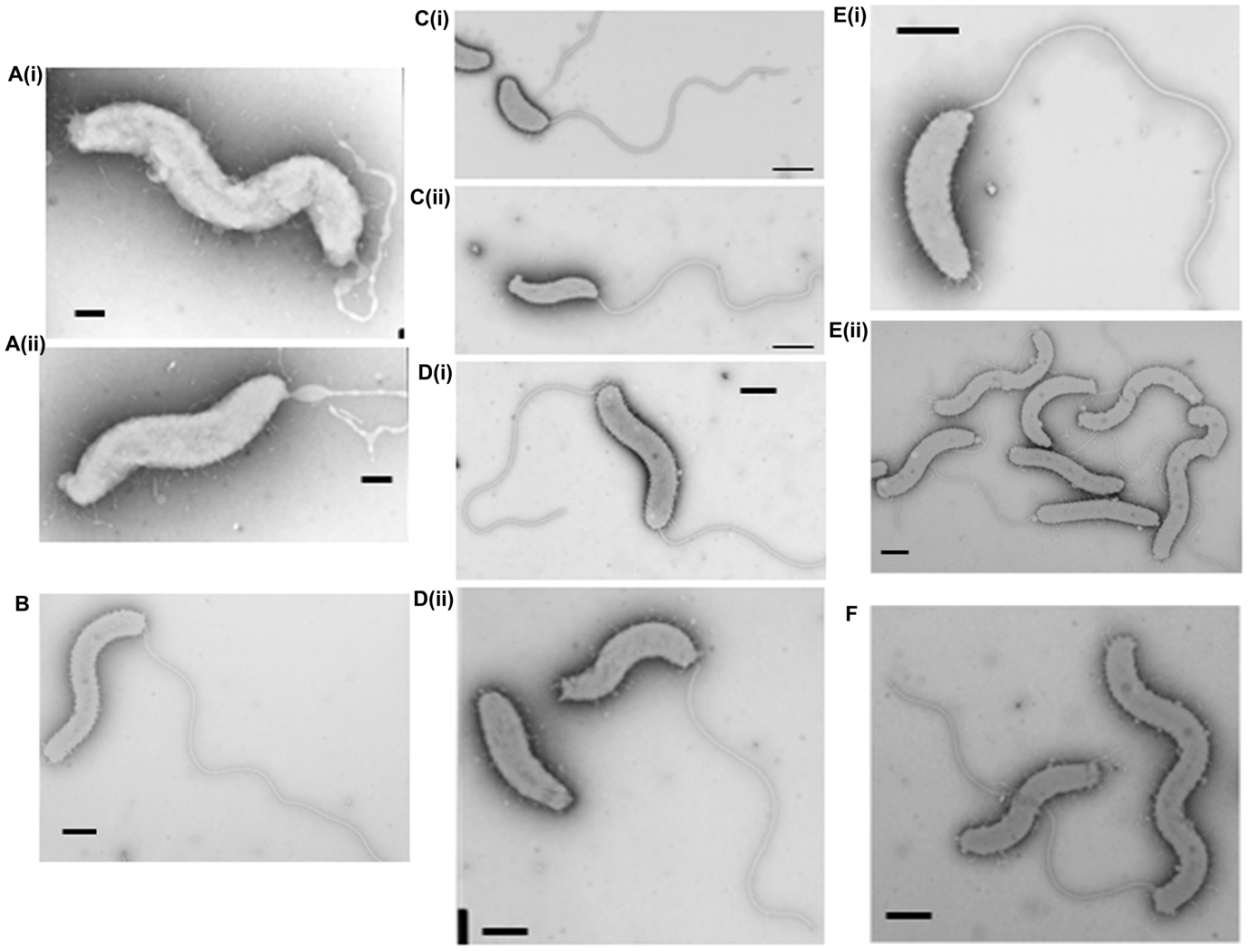
Cell morphology of *dgc* and *cdg* deletion strains revealed by electron microscopy. A(i) and (ii): HI Δ*dgcA* cells showed no functional flagella filaments, only empty membranous flagellar sheath. B: HI Δ*dgcB* cells showing typical cell morphologies. C: (i) Δ*dgcC* HD cells and (ii) typical wild-type HD cells, showing the cell size differences of the Δ*dgcC* mutant. D: (i) rare HI Δ*dgcC* ^sup^ populations have a higher than normal percentage (10%) of bipolarly flagellate cells, (ii) a wild-type *Bdellovibrio* strain HID13 has a mix of cells with no or one polar flagellum. E: (i) HD Δ*dgcD* cells and (ii) HI Δ*dgcD* cells, showing wild-type cell morphology and typical sheathed flagella. Some membrane blebbing was visible on the HI Δ*dgcD* cells but not in significant excess to that seen on other wild-type HI cells. F: the Δ*cdgA* HI cells have typical HI cell morphologies and typical levels of flagellated cells. All cells were stained with 1% PTA (pH 7.0), scale bars = 0.5 µm.

**Figure 4:**
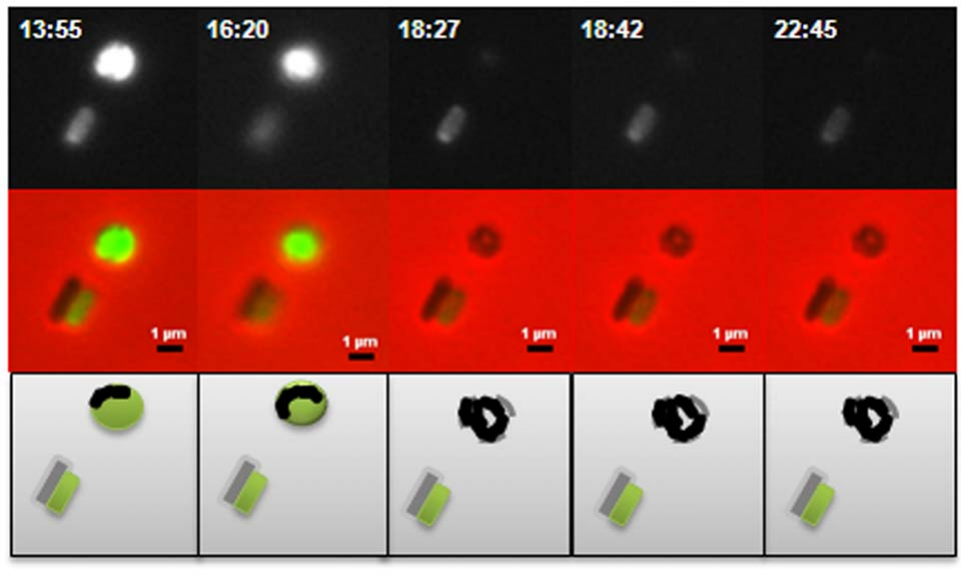
Predatory escape defect of the Δ*dgcA* cells from fluorescent *E. coli* prey. The Δ*dgcA* strain enters prey (not shown) grows inside the bdelloplast, septates and lyses the prey outer membrane, releasing the fluorescence of the prey cell, but does not escape the remnants of the prey, remaining there for over 4 hours, compared to 10 minutes for wild type; scale bars = 1 µm.

**Figure 5:**
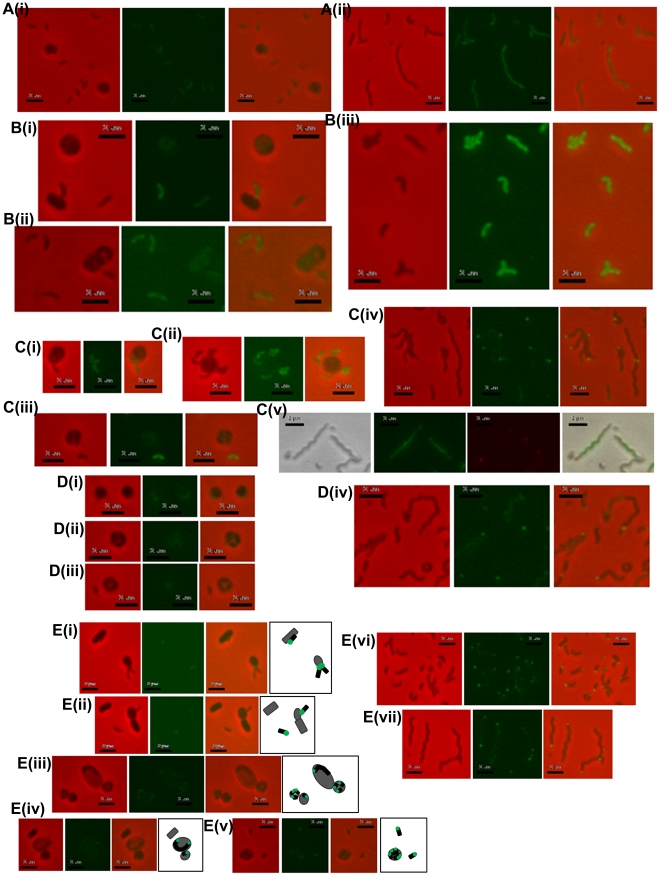
Location of fluorescently tagged Dgc and Cdg proteins in wild-type predatory and axenically growing cells. A: (i) HD wild-type *Bdellovibrio* expressing DgcA-mCherry, showing constitutive low level fluorescence in cells that have invaded prey or which are free swimming; (ii) HI axenic *Bdellovibrio* expressing DgcA-mCherry, again fluorescence is distributed throughout the cells. B: (i and ii) HD wild-type *Bdellovibrio* expressing DgcB-mCherry showing constitutive fluorescence throughout the cell at all points in the predatory lifecycle, in *Bdellovibrio* that have invaded prey or which are free swimming; (iii) HI axenic wild-type *Bdellovibrio* expressing DgcB-mCherry, again fluorescence is seen throughout the cells. C: HD wild-type *Bdellovibrio* expressing DgcC-mCherry showing constitutive fluorescence throughout both the (i) attack-phase cell and (ii) early in predation, but a dissipation of DgcC fluorescence (iii) later in predation and at septation inside prey; (iv) HI axenic wild-type *Bdellovibrio* expressing DgcC-mCherry, most cells contain some bright foci, typically at the poles of the cell, but some in the centre; (v) Dapi-stained HI cells with DgcC-mCherry, green fluorescence is the Dapi-stained DNA, the red foci are the DgcC-mCherry. DgcC-mCherry often forms mid-cell foci in areas without chromosomal DNA, suggestive of location at the potential septal points. D: (i, ii and iii) HD wild-type *Bdellovibrio* expressing DgcD-mCherry showing very weak fluorescence; (iv) HI axenic *Bdellovibrio* expressing DgcD-mCherry, faint fluorescence throughout the cells with some bright foci. E: (i – v) Fluorescent localization of CdgA during the predatory cycle with illustrative diagrams. During intracellular growth and division the CdgA remains at the poles (i, ii and iii) early in the predatory cycle, after prey invasion, the fluorescence is seen only at one pole, (iv) later, when the *Bdellovibrio* is growing (inside prey) from both poles, CdgA is seen at both poles (v) at septation each progeny CdgA-mCherry cell has both a fluorescent and a non-fluorescent pole; (vi and vii) HI cells containing CdgA-mCherry also show polar localization, (vi) smaller cells have a single fluorescent pole whilst (vii) longer cells (part of the natural variation in lengths of HI grown *Bdellovibrio*) typically have fluorescence at both poles, both of which may be active for prey-interaction (Figure S6 in Text S1). All scale bars = 2 µm.

Electron microscopy (Figure 3AI, ii) showed that although a membranous flagellar sheath was made, no functional flagellum was assembled within it. This appearance was reminiscent of a non-motile *fliC3* flagellin mutant previously studied in *Bdellovibrio* [1], [28]. Studies with that *fliC3* mutant had shown it would enter prey cells if applied directly onto them on a solid surface, and could use gliding motility to exit the bdelloplast after septation and lysis [1], [2]. Therefore cells of the Δ*dgcA* mutant were applied to *E. coli* prey (that were constitutively expressing mCherry) on a 1% agarose surface, the prey were invaded and the Δ*dgcA* mutant replicated and septated within them. However, after septation no induction of (flagellar or) gliding motility was seen in the Δ*dgcA* strain and the septated *Bdellovibrio* remained motionless inside the dead “shell” of the prey bdelloplast long after (as long as 120 minutes was measured without any change being observed) the prey mCherry fluorescence was dissipated by the lysis of the bdelloplast (Figure 4). In contrast, wild-type *Bdellovibrio* lyse bdelloplasts and immediately (after 10 minutes), emerge, using gliding motility if on an agarose surface. Thus the deletion of the *dgcA* gene rendered the cells effectively unable to be predatory, as they could not escape the prey remains to search for new prey. A DgcA-mCherry strain was seen to be fluorescent throughout the cell at all times through the predatory cycle (Figure 5Ai), and during Host-Independent growth (Figure 5Aii), not only at times when the cells would be motile (by either flagellar- or gliding- motility).

Complementation of the Δ*dgcA* mutant with a full length but C-terminally mCherry-tagged wild type *dgcA* gene was achieved by single recombination into the chromosome from suicide plasmid pK18*mobsacB*. Exconjugant *Bdellovibrio* from this complementation regained the ability to form plaques on *E. coli* prey lawns (Figure 2Bi) and typically formed 2×0^3^ plaques per conjugation, compared to zero for the parental Δ*dgcA* mutant strain (Figure 2Ai). Gliding was restored to the Δ*dgcA* mutant by this complementation (Figure S4 in Text S1) but interestingly flagellar motility was not. However, we have previously shown that flagellar motility is not required for predation [1]. We were able to observe, by video microscopy, gliding cells of the complemented strain having lysed and escaped from the prey bdelloplasts. Unfortunately the full-length *dgcA* gene without mCherry tag proved difficult to isolate (possibly due to c-di-GMP effects in cloning strains of *E. coli)* so the phenotype of the complemented Δ*dgcA* strain was only discernable from the mCherry tagged *dgcA* gene. We acknowledge that the presence of the mCherry tag may interfere with protein-protein interactions that could be required for full complementation, so we cannot test the flagellar effect. However as gliding motility and predatory growth were restored by this *dgcA-mcherry* complementation, we conclude that the DgcA protein controls gliding motility and thus successful predatory growth on prey bacteria.

### DgcB controls processes essential for *Bdellovibrio* entry into prey

Inactivation of *dgcB* gave a mutant strain which did not form plaques (Figure 2Aii) and which could only be isolated by HI axenic growth (Figure 3B) but which retained expression of wild-type flagella. Some 50% of axenically grown wild type HI *Bdellovibrio* have a flagellum but only 5% of those HI cells swim (in contrast to the 98% motility of predatory *Bdellovibrio* cultures). For the *dgcB* mutant HI cells 50% had flagella and 10–15% of the cells were motile, more than for the wild type HI strains. When 21 cultures, consisting of seven replicates of three independent isolates, of this strain were challenged with prey bacteria in liquid culture, versus 3 cultures of two wild type (WT) *B. bacteriovorus* HI strain controls, predatory invasion and prey bdelloplast formation was seen in the WT *B. bacteriovorus* HI strains after 9 hours and the prey cultures began to clear by prey-lysis. At this point no bdelloplasts were seen for the Δ*dgcB* mutant strains. At 24 hours of incubation there were no prey remaining in the WT HI cultures but the Δ*dgcB* mutant cultures were full of prey bacteria. In 4 of the 21 test cultures of the Δ*dgcB* mutant strains prey killing and bdelloplast formation were seen after an additional 120 hours incubation. In the remaining 17 cultures however, no bdelloplasts were seen despite prolonged further incubation. The 4 presumed suppressor strains were tested by PCR for the presence of the original Δ*dgcB* mutation and this was confirmed.

Repeating this experiment on two different occasions, in a different experimental setting, luminescent prey bacteria [29] were incubated in 96 well Optiplates (Porvair Biosciences) in a BMG luminescent plate reader with 18 different cultures of two different isolates of the Δ*dgcB* mutant (including the isolate that had given presumed suppressor strains before), were incubated for 72 hours. The predator:prey ratio in these latter experiments should have shown clearly, even low-levels (5% of total) of prey-killing by a drop in prey luminescence. No such drop in luminescence was seen in any of the 18 isolates, (data not shown) showing that the phenotype of the Δ*dgcB* mutant is non-predatory, but that rare suppressor mutants may be isolated (these suppressor strains are currently the subject of further study).

A DgcB:mCherry fusion protein expressed in a wild type DgcB background was constitutively brightly fluorescent in the cytoplasm of *B. bacteriovorus* cells whether they were growing predatorily (Figure 5 Bi-ii) or axenically (Figure 5 Biii). As the DgcB protein contains a predicted forkhead domain at the N terminus, we conclude that it may be activated via this domain when its c-di-GMP synthetic properties are biologically appropriate. This activation may be in response to prey sensing.

Complementation of the Δ*dgcB* mutant with the wild type *dgcB* gene was achieved by single recombination into the chromosome from suicide plasmid pK18*mobsacB*. A single complementation conjugation gave rise to 1.7×10^4^ plaques on prey lawns (Figure 2Bii) in stark contrast to the Δ*dgcB* mutant itself which did not form plaques (Figure 2Aii) and had to be cultured axenically as HI strains (and in which only very rare suppressor strains emerged after many days of prey challenge in liquid cultures of those axenically growing HI Δ*dgcB* mutants).

Further evidence that the diguanylyl cyclase activity of the DgcB itself was important for predation came from the conjugation of a GGAAF variant into the Δ*dgcB* mutant instead of the wild type conjugation. The GGAAF plasmid did not restore plaquing ability (Figure 2C) to the Δ*dgcB* strain. Thus we conclude that the diguanylyl cyclase activity of the DgcB protein controls, via c-di-GMP signalling, processes that are required for the invasion of prey bacteria.

### DgcC is required for the conversion of *B. bacteriovorus* to axenic growth

**Figure 6:**
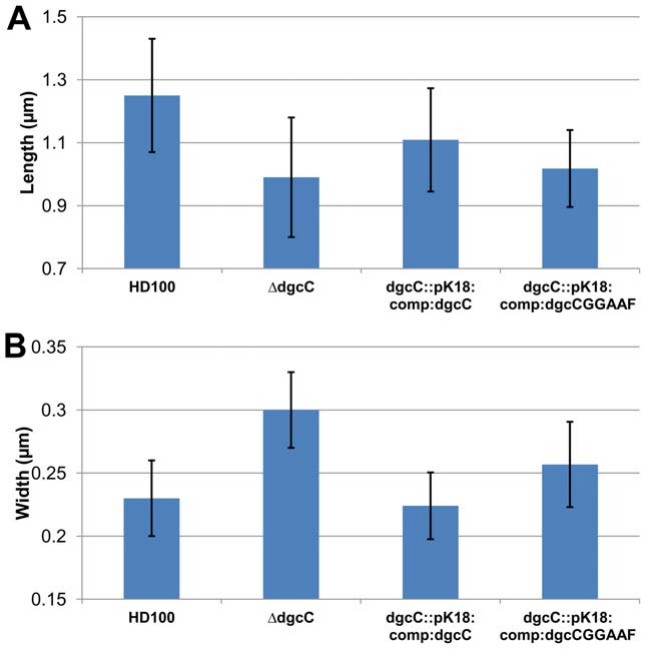
Cell size comparisons of wild-type, Δ*dgcC*, *dgcC* wild-type GGEEF-complemented and *dgc*CGGAAF “complemented” *Bdellovibrio* strains. Histograms show the mean A: length and B: width measured from negatively strained electron microscope images of a minimum of 50 cells per strain. Error bars show one standard deviation above and below the mean. The lengths of each of the mutant and complemented strains was significantly shorter than wild-type (p<0.001) but for the widths, the strain complemented with the wildtype *dgcC* gene which was not significantly wider than wild type, but the mutant strain was significantly (p<0.001) wider; with the *dgcC*GGAAF complemented strain being of intermediate width.

Inactivation of the *dgcC* gene produced attack phase *Bdellovibrio* cells that although normally flagellate, were smaller, but wider (Figure 3Ci) than wild type (Figure 3C ii) (Figure 6). The *dgcC* mutant was still predatory, and invaded and killed *E. coli* prey cells at wild type rates. However, the normal 1 in 10^7^ conversion of predatory *Bdellovibrio* to axenically growing HI *Bdellovibrio* [9] was lost in this mutant and 4.5×10^11^ cells had to be applied to nutrient media to isolate and culture a rare single colony (Figure 3Di), growing axenically. This suggested a secondary mutation was required to allow the Δ*dgcC* mutant to grow axenically. Bacteria from such suppressor strains were frequently (10%) biflagellate, but not markedly different (within the natural variation of HI *Bdellovibrio* from wild-type HI strains) as exemplified by the wild-type strain HID13 (Figure 3Dii).

Fluorescent microscopy of DgcC-mCherry in *B. bacteriovorus* carrying a wild type copy of the *dgcC* gene, showed a cytoplasmic distribution in attack-phase *B. bacteriovorus* (Figure 5Ci) and only faint fluorescence when *Bdellovibrio* was elongating inside prey cells (Figure 5Cii), with fluorescence gone when the predatory cells were septating in the bdelloplast (Figure 5Ciii). Attack-phase cells of the fluorescent DgcC-mCherry strain (which also carried a wild type *dgcC* gene) readily converted (at wild type, 1 in 10^7^ frequency), into axenically growing HI cultures. In these HI cells there were strong fluorescent foci of DgcC-mCherry expression at one pole of each cell (Figure 5Civ) and in 60% of dividing HI cells DgcC foci were seen associated with division points at non-Dapi-staining regions (Figure 5Cv). There were also fainter foci along the length of non-dividing longer HI cells (Figure 5Civ,v). There was no overall cytoplasmic distribution, in contrast to that of the DgcC-mCherry expression in predatory attack-phase cells (which do not divide outside of prey).

We propose that DgcC may be required to regulate predatory cell division and/or cytoskeletal regulation during predatory growth. This is because the Δ*dgcC* progeny produced were shorter and fatter than wild-type (Figure 3Ci, ii, Figure 6). Also it was noteworthy that DgcC-mCherry fluorescence was present when the *Bdellovibrio* were outside prey or in the early stages of invasion (Figure 5Ci,ii), but that it dissipated at septation (Figure 5Ciii)., suggesting DgcC turnover at septation. Wild-type attack-phase *Bdellovibrio* cannot replicate outside prey, due to an unknown control mechanism [7]. It is interesting, in support of this, that the conversion to axenic growth mode of *Bdellovibrio* was abolished in the DgcC mutant. Axenic and predatory division of *Bdellovibrio* does not have to use a completely overlapping set of cell division proteins, as the *Bdellovibrio* genome is large, and as predatory division is by synchronous septation of a long filament, yet in axenically growing *Bdellovibrio* budding events (of single cells from the tip of a long filament), binary fission events (of shorter serpentine HI cells) and other division patterns are seen.

One gene, *Bd0108*, at the *hit* locus has been reported by others as a hot spot for mutations which affect the ability of *Bdellovibrio* to convert from predatory to axenic growth [9], [10], [30]. Despite this the sequence of the *Bd0108* gene in the Δ*dgcC* strain was found to be wild type, in addition the sequence of *Bd0108* in the rare (1 in 4.5×10^11^) “HI suppressor of Δ*dgcC*” that grew was also wild type.

Complementation of the Δ*dgcC* mutant with the wild type *dgcC* gene was achieved by single recombination into the chromosome from suicide plasmid pK18*mobsacB*. Exconjugants from this complementation were predatory, as was the original Δ*dgcC* mutant strain, and the cells returned to the wild-type cell width dimension of 0.23 µm and almost returned to the wild-type length of 1.25 µm (Figure 6). However when a version of the *dgcC* gene, encoding GGAAF instead of wild-type GGEEF (to inactivate the catalytic site of the protein) was used in identical conjugation experiments, the exconjugant strains were not fully complemented in terms of cell size (Figure 6), showing intermediate size distributions of length and width.

The normal 1 in 10^7^ conversion of wild type predatory *Bdellovibrio* to axenically growing HI *Bdellovibrio* (Figure 2Di) was restored to the Δ*dgcC* mutant by wild type *dgcC* complementation (Figure 2Dii) and colonies of axenically growing *Bdellovibrio* were readily isolated from nutrient media plates. Further experiments will determine whether the GGAAF encoding version of the *dgcC* plasmid restores the normal HI conversion rate.

### DgcD protein has no obvious role in *B. bacteriovorus* behaviour

In contrast to the other GGDEF genes, an in-frame deletion of the *dgcD* gene did not cause an observable phenotype in the conditions that we measured and the mutant strains could be cultured in both HD (Figure 2Aiii) and HI growth modes without alteration in growth rates. Introduction of the wild type *dgcD* gene had no effects on growth (Figure 2Biii). They also retained wild type morphology and motility (Figure 3Ei, ii). Bright fluorescent foci of DgcD-mCherry were seen (Figure 5D iv) in wild type HI *Bdellovibrio*, but very weak constitutive fluorescence only, was seen in HD cells (Figure 5 D I,ii,iii). Sequencing of another *B. bacteriovorus* isolate in our laboratory (in preparation) has shown that while the other 4 GGDEF genes are conserved across strains, that *dgcD* is not. Due to the lack of a significant phenotype for the *dgcD* deletion and the lack of conservation among *Bdellovibrio* strains, we did not investigate the role of DgcD any further.

### CdgA organizes processes at the *Bdellovibrio* “nose” that are crucial to rapid prey invasion

Exconjugants with an in-frame deletion of the *cdgA* gene were not predatory and did not form plaques on prey lawns (Figure 2A iv) and Δ*cdgA Bdellovibrio* could only be isolated in HI axenically grown plate cultures (Figure 3F). However, when the Δ*cdgA* mutant was offered to prey cells in mixed liquid cultures, prey invasion and bdelloplast formation did proceed, but slowly, i.e. 40–90 minutes for Δ*cdgA* versus 30–40 minutes for wild-type HD strains (Figure S5 in Text S1). This slow predation resulted in liquid cultures taking two days to clear the majority of prey cells as compared to around 16 hours for wild-type strains. This slow prey invasion, likely accounted for the failure of the Δ*cdgA* mutant to be efficiently cultured predatorily on plates of prey lawns when originally isolated. Furthermore, when the Δ*cdgA* mutant, that had been growing slowly predatorily in liquid cultures, was returned to prey lawns on agar plates, they again failed to form plaques. Complementation of the Δ*cdgA* mutant with the wild type *cdgA* gene was achieved by single recombination into the chromosome from suicide plasmid pK18mobsacB. Exconjugants from this complementation gave rise to >10^5^ plaques per conjugation, counted on dilution plates of prey lawns (Figure 2B iv) in contrast to none from matched numbers of the original Δ*cdgA* strain (Figure 2A iv). Invasion of prey cells by the complemented strain, previously grown on prey, was found to occur after 30–40 minutes (a wild type speed) by timelapse microscopy (Figure S5 in Text S1).

Fluorescent protein localization of CdgA-mCherry, in a wild type CdgA-expressing *B. bacteriovorus* HD100, showed that it was expressed at the non-flagellar pole or prey-interacting “nose” of the predatory *B. bacteriovorus* cells and that this expression was seen when the *Bdellovibrio* were attached to the prey cell at the point of invasion (Figure 5E i, ii). After invasion a single fluorescent polar focus of CdgA-mCherry persisted whilst the *Bdellovibrio* began to elongate, initially from this single pole. Then a second CdgA-mCherry focus developed at the second pole - during the period in which the *Bdellovibrio* grows via bipolar elongation (Figure 5E iii, iv); [6]). Upon septation of the predatory *Bdellovibrio*, within the prey bdelloplast, a single bright CdgA-mCherry focus was seen at one pole of each septated *Bdellovibrio* cell, prior to release from prey (Figure 5E v).

In axenically-growing, longer, HI cells polar and sometimes bipolar expression of this protein was also seen (Figure 5E vi, vii). This pattern correlates with video-microscopy observations we have made where some unusually long HI *Bdellovibrio* wild-type cells, found by chance with each pole adjacent to a prey cell, simultaneously invade both prey cells at once. In such unusually long HI *Bdellovibrio* (which do not have the usual polar flagellum), it seems that each pole can be competent for prey entry (Figure S6 in Text S1).

Among mutations in the c-di-GMP signalling proteins, the Δ*dgcB* and Δ*cdgA* mutations were the only ones that impaired predatory growth, specifically at the prey entry stage, which suggests that DgcB and CdgA belong to the same regulatory cascade, where DgcB signals, via c-di-GMP synthesis, to CdgA. A more severe phenotype of the *dgcB* deletion versus the *cdgA* deletion implies that DgcB signals via more than a single target. The DgcB targets likely regulate activity/expression of host hydrolytic and prey modifying/degrading proteins involved in penetration of prey bacteria. It appears that diguanylyl cyclase activity of DgcB is activated via an encounter with prey, possibly via the forkhead domain of DgcB. Using the DgcB-mCherry fusion we determined that DgcB is apparently expressed at relatively high levels and located throughout the cell.

### Analysis of c-di-GMP levels in axenically grown Δ*dgc* and Δ*cdg* mutant strains

The method described by Bobrov and co-workers [31] was used to determine the c-di-GMP levels in pure *Bdellovibrio* cells from the axenically grown Δ*dgcA, B* and Δ*cdgA* mutant HI strains, versus matched wet cell weights of wild type HI controls. A similar analysis was not possible for predatory strains due to possible contamination with prey-derived c-di-GMP. The mean level of c-di-GMP for wild type axenic HI *B. bacteriovorus* strains HID13 and HID26 was 1.4+/−0.1nM/mg wet weight of cells. For the Δ*dgcA* strain this value was considerably lower at 0.4nM/mg wet weight of cells, for the Δ*dgcB* strain the value was 1.5nM/mg wet weight of cells and for the Δ*cdgA* strain the value was slightly higher at 2.0nM/mg wet weight of cells. Thus the absence of the DgcA protein noticeably reduced the extractable c-di-GMP content of the *B. bacteriovorus* cells, consistent with the diguanylyl cyclase activity of DgcA. The absence of the CdgA protein slightly increased the extractable c-di-GMP content but the absence of the DgcB protein did not cause a significant difference to extractable c-di-GMP levels. The latter result is not surprising given the expectation that DgcB is activated when the *Bdellovibrio* are in contact with, or the immediate proximity of prey (which was not the case in the axenically grown cells used here.

### Summary

**Figure 7:**
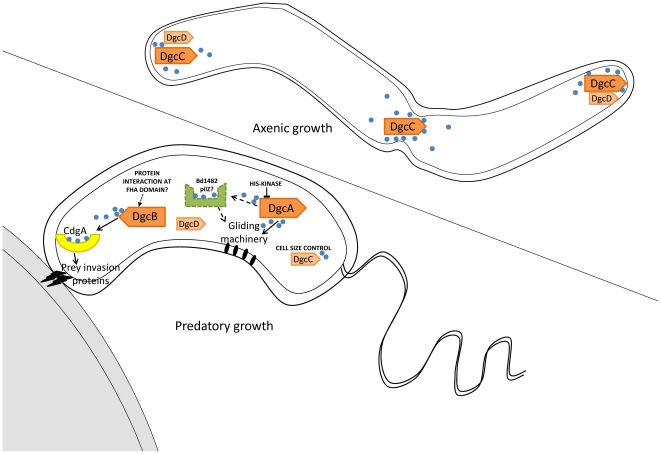
Summary of GGDEF domain protein activities in *B. bacteriovorus* HD100 lifestyles. Arrows with solid lines summarise regulatory activities discovered by analysis of phenotypes of DgcA,B,C and CdgA mutant strains. Dotted arrows and lines indicate pathways inferred by association of a PilZ protein. Positions of DgcC and DgcD in the axenically growing *Bdellovibrio* were informed by mCherry tagging. The position of CdgA at the prey-interacting pole of the predatory *Bdellovibrio* was informed by mCherry tagging, with the remaining DgcA and DgcB mCherry proteins being found to be cytoplasmic in these cells.

In summary, (Figure 7) we have found that each of 4 conserved GGDEF proteins in *B. bacteriovorus* HD100 contributes non-overlapping regulatory controls to different aspects of the predatory or axenic life cycles of this bacterium, and that these can be seen in single GGDEF gene mutants with the remaining GGDEF genes intact. We found that DgcB controls predatory invasion of prey bacteria, by signalling to c-di-GMP receptors, probably including CdgA, a degenerate GGDEF protein located at the prey-interacting “nose” of *Bdellovibrio*. As DgcB may receive information that indicates proximity or attachment to prey being sensed by the *Bdellovibrio*, we are now searching for the signals that activate DgcB. This will allow us to understand signals that “tell” *Bdellovibrio* that prey are present and aid the targeting of Gram-negative pathogens for destruction in anti-infective settings.

DgcA controls gliding motility (Figure 7) which is required for the *Bdellovibrio* to exit the exhausted prey debris after predation and to move off to regions where new prey encounters would be possible [1], [2]. To our knowledge this is the first case where c-di-GMP controls gliding motility, whereas c-di-GMP control of flagellar motility is better understood in other bacteria. As only gliding motility and predation, not flagellar synthesis, were found to be restored by the DgcA*-*mCherry complementation experiment, it may be that DgcA does not regulate flagellar synthesis in *B. bacteriovorus* HD100. DgcA has a response regulator domain at the N terminus and thus has some similarity to WspR which controls flagellar biogenesis versus pellicle formation in *Pseudomonas* [32], [33]. Flagellar biogenesis is not however required for *B. bacteriovorus* predation [1] and further studies on DgcA will define whether it also regulates flagellar biogenesis. We do note that, at the 5′ end of one of 4 operons of gliding motility genes of *B. bacteriovorus* [2], there is a gene, Bd1482, encoding a PilZ domain protein, which may be a candidate for receiving signals from DgcA to effect gliding and thus we have tentatively labelled this (Figure 7) as a candidate to receive c-di-GMP signals from DgcA. Gliding will be particularly important in the predation of pathogen biofilms where *Bdellovibrio* have been shown to be effective [34].

DgcC controls the transition between the predatory “attack phase” of *Bdellovibrio* when it is “locked into” a non-replicative phase, hunting prey; and the replicative, axenic, growth phase, on protein rich media. This is a mysterious transition [10], [35]. It is noteworthy that DgcC-mCherry forms foci in growing axenic cells and we hope that studying any proteins with which DgcC forms a complex may illuminate this control of non-predatory replication. The DgcC deletion mutant is the gateway to producing a useful, obligately predatory *Bdellovibrio* for application as a self-limiting living antibiotic.

We have revealed the impressive c-di-GMP intellect of this tiny bacterium and how it is employed in its unique intra-bacterial niche, and we can now see how this can be manipulated to use it as an anti-infective [18]. Studying the PilZ receptor domain proteins of *Bdellovibrio*, of which there are 15 [36], will dissect further, the stages by which predatory and axenic growth processes are regulated, and will reveal the cyclase- receptor relationships.

This study has contributed to our understanding of specificity of c-di-GMP signalling pathways; something where different models have been proposed from workers studying other bacteria. Some have suggested that little specificity exists between diguanylyl cyclases and their targets [37]; with all expressed diguanylyl cyclases contributing to the intracellular c-di-GMP pool that is sensed by all c-di-GMP receptors or targets. In an alternative, “c-di-GMP cloud” model [38], each diguanylyl cyclase has a specific target; little crosstalk exists between different c-di-GMP signalling pathways because c-di-GMP bursts are localized, and the spillover is prevented by c-di-GMP phosphodiesterases. Several studies have supported the later model by data showing that intracellular levels of c-di-GMP do not necessarily correlate with the observed phenotypes, that c-di-GMP is produced at unique cellular locations, and most directly, specific c-di-GMP targets were linked to individual diguanylyl cyclases [39]-[43]. However, *B. bacteriovorus* is the first bacterium in which the strikingly different phenotypes of the diguanylyl cyclase mutants shows an exclusive cyclase- c-di-GMP target(s) relationship. It is ironic that the task of proving the existence of c-di-GMP signalling pathway specificity has fallen onto one of the tiniest bacteria, where diffusion rates could have been envisioned to make c-di-GMP spillover unavoidable. The small size of *Bdellovibrio* cells makes the exclusivity point most convincing. While some bacteria may have ‘general purpose’ diguanylyl cyclases, and activation of parallel c-di-GMP targets is perhaps advantageous for some organisms, under certain circumstances; specific c-di-GMP signalling from discrete diguanylyl cyclases is clearly at play in *Bdellovibrio* control pathways, and as this specificity occurs in such small bacteria; it should also be considered in others.

## Materials and Methods

### Strains and growth conditions

*Bdellovibrio* and *E. coli* strains are listed in Table S1 (in Text S1). Predatory *B. bacteriovorus* strains were routinely grown in Ca/HEPES with *E. coli* S17-1 as prey as previously described [1], [44]. Host-Independent (HI) *Bdellovibrio* were isolated and grown in PY broth as previously described [44], [45]. Kanamycin (25 µg ml^−1^ for *E. coli* and 50 µg ml^−1^ for *Bdellovibrio*), ampicillin (50 µg ml^−1^) and isopropyl-β-D-1-thiogalactopyranoside (IPTG; 200 µg ml^−1^ for induction of fluorescence in *E. coli*) were used where appropriate.

### Gene deletion and complementation in *Bdellovibrio*

Chromosomal deletions of the *dgcA*, *dgcB*, *dgcD* and *cdgA* reading frames (*Bd0367*, *Bd0742*, *Bd3766* and *Bd3125*) were created using a modified version of previously described methods [27], [46]. Deletion of the *dgcC* (*Bd1434*) ORF and replacement with a kanamycin resistance cassette was initially performed using a modified version of that described by Lambert and co-workers [29] and later followed up, to verify the cell size phenotype with the same *dgcC* chromosomal deletion using the silent deletion method as used for the four other genes above. Complementation analyses for *dgcA*, *dgcB*, *dgcD* and *cdgA* deletion strains used single recombination of either full-length mcherry tagged (for *dgcA*) or wild type *dgc or cdg* genes (for the other strains) into the *Bdellovibrio* chromosome from suicide plasmid pK18mobsacB. For strains *dgcB* and *dgcC only,* GGAAF expressing (rather than wild type GGDEF or GGEEF) genes were used in single recombination experiments to test the function of the GGDEF domain. (Wild type CdgA does not have a conventional GGDEF domain so was excluded). Construction of each of the mutant and complemented strains is described fully in Text S1.

### Electron Microscopy (EM)

*Bdellovibrio* deletion strains were assayed for morphological changes by EM, after growth (both predatory and axenic) under standard conditions. Cells were stained with 1% phosphotungstic acid (PTA; pH 7.0) and imaged with a JEOL 1200Ex electron microscope at 80 kV. Replicate cultures were grown independently on different days and flagellar patterns and cell morphologies were determined from 45–50 cells observed each time.

### Assays for predatory and axenic growth capabilities

Deletion mutants of *dgcA*, *dgcB* and *cdgA* could only be isolated using axenic (HI) growth. HI deletion strains were added in excess to prey in Ca/HEPES buffer and monitored by microscopy for signs of prey entry and replication at several time-points during prolonged incubation over multiple days (up to 10 days for Δ*dgcB*; whilst wild-type HI strains [HID2, HID13 and HID26 [47], chosen for morphological similarities, see important considerations in Text S1] completed predation when checked after 24 hours of incubation). Both the Δ*dgcA* and Δ*cdgA* mutants were seen to enter prey within the first 24–48 hours of incubation in these liquid conditions and these were further analysed by time-lapse microscopy (described below). Cells from prolonged incubation of Δ*dgcB* mutant strains with prey were re-confirmed to be Δ*dgcB* by PCR analysis. Deletion mutants of both *dgcC* and *dgcD* could be readily obtained from screening predatory cultures, to check whether these strains could grow axenically they were “turned HI” by filtration of attack-phase cells through a 0.45 µm filter to remove any remaining prey, and plating onto rich Peptone-Yeast Extract media [45]. The number of attack-phase cells of the Δ*dgcC* mutant had to be multiplied 1000 –fold (to 4.5×10^11^) to obtain a single colony on the PY media. Complemented strains were tested by the same methods.

### C-di-GMP activity measurements

The diguanylyl cyclase assays *in vitro* were performed by measuring the rate of GTP conversion into c-di-GMP by MBP-Dgc fusion proteins. GTP was added to the purified enzyme solution following the protocol by Ryjenkov et al. [48]. Each dignuanylyl cyclase reaction contained 5 micromoles of the MBP-Dgc fusion proteins. The equilibrium dialysis measurements of c-di-GMP binding to CdgA were performed using 10 micromole of MBP-CdgA and varying concentrations of c-di-GMP as described elsewhere [26]. Nucleotide separation and quantitation was accomplished by HPLC as described by Ryjenkov et al. [48].

### Extractable c-di-GMP determinations for *Bdellovibrio* cells

The method of Bobrov [31] was used to determine the extractable levels of c-di-GMP in axenically grown *Bdellovibrio cells.* with analysis using liquid chromatography tandem mass spectrometry carried out by Lijun Chen and Bev Chamberlin at the Mass Spectrometry Core of RTSF (Research Technology Support Facility) at Michigan State University USA Axenically growing HI *Bdellovibrio* strains (mutant and wild type HI) were grown in 50ml of PY broth from a starting 0.2 OD_600nm_ until they reached a final 0.6 OD_600nm_. Cells were then pelleted by centrifugation, the wet weights were determined and matched; they were frozen in liquid nitrogen and then processed using Bobrov's method and extraction buffer (40% methanol 40% acetonitrile in 0.1N formic acid) which was later neutralised with NH_4_HCO_3_. The levels of extractable c-di-GMP in the cell extracts were compared to known added standards of pure c-di-GMP.

### Video microscopy of events in the predatory growth cycle and gliding

Time-lapse back-lit microscopy was performed as described fully by Fenton and co-workers [6], with the exception that HI *Bdellovibrio,* to be tested for predatory capacity, were grown overnight in PY broth and then diluted to a starting OD_600nm_ of 0.75 before addition to prey in the infection culture.

### Localisation of fluorescently tagged proteins

*Bdellovibrio* strains containing GGDEF protein-mCherry fusions, in a wild-type GGDEF gene background, were constructed using a modification of the method described by Fenton and co-workers [49], construction of each tag is described fully in Text S1. Resulting strains were imaged using a Nikon Eclipse E600 epifluorescence microscope with a 100x objective lens and an hcRED filter block (excitation 550 to 600 nm; emission 610 to 665 nm) in conjunction with a Hamamatsu Orca ER camera and the Simple PCI software (version 5.3.1 Hamamatsu). Gliding motility was assayed by timelapse microscopy as in Lambert and co-workers [2].

### Genes studied

Bd0367 dgcA GenBank ID: NP_967362.1

Bd0742 dgcB GenBank ID: NP_967706.1

Bd1434 dgcC GenBank ID: NP_968330.1

Bd3125 cdgA GenBank ID: NP_969891.1

Bd3766 dgcD GenBank ID: NP_970474.1

## Supporting Information

Text S1 SOM text.
(DOC)
