# Supplementary material for: Discrete Cyclic di-GMP-Dependent Control of Bacterial Predation versus Axenic Growth in Bdellovibrio bacteriovorus
Source: PLoS Pathog. 2012 Feb 2;8(2):e1002493. doi: 10.1371/journal.ppat.1002493 (PMC3271064; doi:10.1371/journal.ppat.1002493)

**Text S1 (Supporting online material)**

**Construction of gene deletion mutants in *Bdellovibrio*.** To produce chromosomal gene deletions of *dgcA*, *dgcB*, *dgcD* and *cdgA*, a modified method, related to those of Roschanski and co-workers (1) and Steyert and co-workers (2) was used. Approximately 1 Kb of upstream and downstream DNA from each gene was PCR amplified and fused together (using primers given in Table S2) such that the first two amino acids and the last 4 amino acids of the otherwise deleted ORF remained, and a restriction enzyme site was introduced after the stop codon. These constructs were then cloned into the suicide vector pK18*mobsacB* which was conjugated into *B. bacteriovorus* HD100 from the donor *E. coli* strain S17-1 (3). Resulting recombinant *Bdellovibrio* merodiploid strains were obtained using kanamycin selection (at 50 µg ml^-1^). To screen for predatory deletion mutants, the merodiploid strains were routinely cultured in Ca/HEPES with *E. coli* S17-1 prey, without antibiotic selection, every 24 hours in standard predatory cultures (4). They were then plated onto double-layer overlay plates on lawns of prey, if individual plaques arose cultures from these were screened by PCR for evidence of the second cross-over event occurring and the deletion of the gene of interest. To rescue any non-predatory mutants that would not form plaques, kanamycin-resistant merodiploid strains were “turned HI” by standard methods (5) immediately after the exconjugants first grew. They were plated onto Peptone-Yeast Extract agar plates without antibiotic selection. Resulting HI colonies were screened for kanamycin sensitivity, and those found to be sensitive to kanamycin were screened by colony PCR for gene deletion. If only HI gene deleted strains could be obtained these were further cultured and tested for predatory capacity as in the Materials and Methods.

The initial *dgcC* deletion strain was created using an insertion-deletion method. As before, 1 Kb of upstream and downstream flanking DNA was previously amplified and fused together, introducing an *EcoRV* restriction enzyme site immediately after the stop codon of *dgcC*. The kanamycin resistance cassette from pUC4K was introduced into this site, then the combined construct was cloned into the suicide vector pSET151. This was conjugated into HD100 as described above, and resulting predatory isolates were screened by PCR for evidence of deletion and replacement of *dgcC*. Subsequently, due to the need for a ‘silent’ chromosomal gene deletion for use in complementation assays, a strain carrying a chromosomal deletion of the *dgcC* gene was also created using the same method (using the suicide vector pK18*mobsacB*) as described above for the other genes. All deletion strains were confirmed by Southern blotting, sequencing and by RT-PCR using RNA derived from mutant strains to confirm the lack of transcript of the deleted gene.

**Protein-mCherry fusion construction.** The DNA encoding each ORF was PCR amplified from genomic DNA using the primers in Table S2 which were designed to introduce either a *ScaI* or *EcoRI* restriction site at the N-terminus of the gene, a *KpnI* site at the C-terminus, and amplified all but the stop codon of each gene. The amplified genes were then fused at the 3’ end to give an mCherry monomeric fluorescent protein (6), fused in-frame with each ORF with a short linker sequence encoding the amino acids GTSS in between. These were then cloned into the suicide vector pK18*mobsacB*, and conjugated into wild-type *B. bacteriovorus* HD100, which integrated into the genome of *Bdellovibrio* at the site of the original ORF (7). The resulting fluorescent strains were selected for by addition of kanamycin (50 µg ml^-1^) to maintain the integrated plasmid. The genome of each of these strains contained the protein-mCherry fusion expressed from the native promoter of the respective gene, and a promoter-less copy of the wild-type gene following the integrated pK18*mobsacB* plasmid.

**Complementation of gene deletion mutants in *Bdellovibrio*.** Three different complementation strategies were developed for this study. Each used the suicide vector pK18*mobsacB* in a single crossover into the *Bdellovibrio* chromosome. For the *dgcA* mutant, complementation was achieved using a C-terminal mCherry fusion protein (the untagged full length gene seemed to have deleterious effects on bacterial cloning hosts) . Using the protein-mCherry fusion construct above, a 1kb region of 5’ flanking genomic DNA (amplified using the primers in Table S2) was added to the 5’ end of the gene fusion resulting in a construct that contained the protein-fusion under the control of the native *Bdellovibrio* promoter that would recombine into the genome at the site of gene deletion, giving expression of the gene fusion at native wild-type *Bdellovibrio* levels. For *dgcB*, *dgcC*, *dgcD* and *cdgA*, the genes, along with 1kb of 5’ flanking genomic DNA and 250bp of 3’ flanking DNA were amplified from HD100 genomic DNA (using primers in Table S2) and cloned into pK18*mobsacB*. For *dgcB* and *dgcC*, similar constructs which included an altered version of the gene resulting in a protein sequence change from GGEEF to GGAAF removing the catalytic c-di-GMP synthetic site of each protein were also created. Each construct was conjugated into the respective gene deletion mutant *Bdellovibrio*, recombinants were selected for by addition of kanamycin (50 µg ml^-1^) to maintain the integrated plasmid, which re-integrated into the chromosome at the site of the original gene deletion.

**Important considerations when choosing axenically growing wild-type *Bdellovibrio* control strains for assaying predatory and axenic growth phenotypes.** Axenically growing, Host-Independent (HI) *Bdellovibrio* have a (published) natural variation in morphology and predatory efficiency (8-10). We used three different axenically-growing wild-type HI strains derived from *B. bacteriovorus* HD100 as wild-type comparisons, strains HID2, HID13 and HID26 (11). Some HI wild-type strains, such as HID2, have a predominance of shorter (1-2 µm long) flagellate cells, and others, such as HID26, have a predominance of longer (2-10 µm long) cells, and strain HID13 is intermediate (11). We used electron microscopy for morphological comparisons with these controls so we could see any true phenotypic changes from within the natural morphological diversity of HI *Bdellovibrio*. Each of these wild-type HI strains of *B. bacteriovorus* HD100 do retain the ability to convert back to predation if prey cells are offered, so these were used as a fair comparison in “prey challenge” experiments to test whether the GGDEF mutant strains retained any predatory capacity (5, 11).

***In vitro* diguanylyl cyclase assays and *in vitro* c-di-GMP binding.** The *dgcA*, *dgcB*, *dgcC* and *cdgA* genes and the fragment encoding the GGDEF domain from DgcD (GGDEF_DgcD_) were cloned in vector pMAL-c2x (NEB Biolabs) using primers and restriction sites shown in Table S2. The MBP-fusion proteins were overexpressed in *E. coli* DH5α following a standard overexpression protocol (12). The partially optimized conditions for expression of the soluble fusion proteins were as follows: MBP-DgcA (18°C; 15 h); MBP-DgcB (37°C; 2.5 h); MBP-DgcC (28°C; 5.5 h); MBP-GGDEFDgcD (37°C; 2.5 h); MBP-CdgA (18°C; 15 h). In all instances, proteins were induced by addition of 0.5 mM IPTG (final concentration) to the exponentially grown culture at optical density A600 ~0.6. Proteins were purified using amylose affinity chromatography as recommended by the manufacturer (NEB Biolabs). Protein purity was verified by SDS-PAGE. The diguanylyl cyclase assays and HPLC separations of nucleotides were performed as described earlier (12). The equilibrium dialysis measurements of c-di-GMP binding to CdgA were performed as described elsewhere (13).

***In vivo E. coli* assays for diguanylyl cyclase activity and c-di-GMP binding.** Late log-phase cultures of the *E. coli* MG1655 strains containing the plasmids for expressing the MBP fusion proteins created for the in vitro assays were concentrated, and 3 µl was inoculated onto soft agar plates containing 0.25% agar, 1% tryptone and 0.5% NaCl. Images were taken after 6 h at 37°C. Swarm sizes were compared to give an estimation of diguanylyl cyclase activity or c-di-GMP binding of each of the fusion proteins.

**References.**

1. Roschanski N, Klages S, Reinhardt R, Linscheid M, & Strauch E (2011) Identification of genes essential for prey-independent growth of *Bdellovibrio* *bacteriovorus* HD100. *J Bacteriol* 193(7):1745-1756.

2. Steyert SR & Pineiro SA (2007) Development of a novel genetic system to create markerless deletion mutants of *Bdellovibrio* *bacteriovorus*. *Appl Environ Microbiol* 73(15):4717-4724.

3. Lambert C, Smith MC, & Sockett RE (2003) A novel assay to monitor predator-prey interactions for *Bdellovibrio* *bacteriovorus* 109 J reveals a role for methyl-accepting chemotaxis proteins in predation. *Environ Microbiol* 5(2):127-132.

4. Lambert C & Sockett RE (2008) Laboratory maintenance of *Bdellovibrio*. *Curr Protoc Microbiol* Chapter 7:Unit 7B 2.

5. Evans KJ, Lambert C, & Sockett RE (2007) Predation by *Bdellovibrio* *bacteriovorus* HD100 requires type IV pili. *J Bacteriol* 189(13):4850-4859.

6. Shaner NC, et al. (2004) Improved monomeric red, orange and yellow fluorescent proteins derived from *Discosoma* *sp*. red fluorescent protein. *Nat* *Biotechnol* 22(12):1567-1572.

7. Fenton AK, Lambert C, Wagstaff PC, & Sockett RE (2010) Manipulating each MreB of *Bdellovibrio* *bacteriovorus* gives diverse morphological and predatory phenotypes. *J Bacteriol* 192(5):1299-1311.

8. Barel G & Jurkevitch E (2001) Analysis of phenotypic diversity among host-independent mutants of *Bdellovibrio* *bacteriovorus* 109J. *Arch* *Microbiol* 176(3):211-216.

9. Thomashow MF & Rittenberg SC (1979) Descriptive biology of the bdellovibrios. *Developmental biology of prokaryotes*, ed Parish JH (University of California Press), 9th Ed.

10. Wurtzel O, Dori-Bachash M, Pietrokovski S, Jurkevitch E, & Sorek R (2010) Mutation detection with next-generation resequencing through a mediator genome. *PLoS* *One* 5(12):e15628.

11. Lambert C, Chang CY, Capeness MJ, & Sockett RE (2010) The first bite--profiling the predatosome in the bacterial pathogen *Bdellovibrio*. *PLoS* *One* 5(1):e8599.

12. Ryjenkov DA, Tarutina M, Moskvin OV, & Gomelsky M (2005) Cyclic diguanylate is a ubiquitous signaling molecule in bacteria: insights into biochemistry of the GGDEF protein domain. *J Bacteriol* 187(5):1792-1798.

13. Ryjenkov DA, Simm R, Romling U, & Gomelsky M (2006) The PilZ domain is a receptor for the second messenger c-di-GMP: the PilZ domain protein YcgR controls motility in enterobacteria. *J Biol Chem* 281(41):30310-30314.

14. Simon R, Preifer U, & Puhler A (1983) A broad host range mobilisation system for in vivo genetic engineering: transposon mutagenesis in gram negative bacteria. *Biotechnology* 9:184-191.

15. Hanahan D (1983) Studies on transformation of Escherichia coli with plasmids. *Journal of Molecular Biology* 166(4):557-580.

16. Rogers M, Ekaterinaki N, Nimmo E, & Sherratt D (1986) Analysis of Tn7 transposition. *Molecular and General Genetics* 205(3):550-556.

17. Fenton AK, Kanna M, Woods RD, Aizawa SI, & Sockett RE (2010) Shadowing the actions of a predator: backlit fluorescent microscopy reveals synchronous nonbinary septation of predatory *Bdellovibrio* inside prey and exit through discrete bdelloplast pores. *J Bacteriol* 192(24):6329-6335.

18. Blattner FR, et al. (1997) The complete genome sequence of *Escherichia* *coli* K-12. *Science* 277(5331):1453-1474.

19. Rendulic S, et al. (2004) A predator unmasked: life cycle of *Bdellovibrio* *bacteriovorus* from a genomic perspective. *Science* 303:689-692.

20. Stolp H & Starr MP (1963) *Bdellovibrio* *Bacteriovorus* Gen. Et Sp. N., a Predatory, Ectoparasitic, and Bacteriolytic Microorganism. *Antonie* *Van* *Leeuwenhoek* 29:217-248.

21. Schafer A, et al. (1994) Small mobilizable multi-purpose cloning vectors derived from the *Escherichia* *coli* plasmids pK18 and pK19: selection of defined deletions in the chromosome of *Corynebacterium* *glutamicum*. *Gene* 145(1):69-73.

22. Bierman M, et al. (1992) Plasmid cloning vectors for the conjugal transfer of DNA from *Escherichia* *coli* to *Streptomyces* spp. *Gene* 116(1):43-49.

23. Finn RD, et al. (2008) The Pfam protein families database. *Nucleic Acids Research* 36(suppl 1):D281-D288.

**Table S1.** Bacterial strains and plasmids.

| **Strain or Plasmid** | **Genotype or description** | **Reference** |
| --- | --- | --- |
|  | *Escherichia coli* |  |
| S17-1 | *thi,pro,hsdR*^-^,*hsdM*^+^,*rec*A; integrated plasmid RP4-Tc::Mu-Kn::Tn*7*; used as donor for conjugating plasmids into *Bdellovibrio* | (14) |
| DH5α | F′ *endA1* *hsdR17*(r_k_^–^m_k_^–^) *supE44 thi-1* *recA1 gyrA* (Nal^r^) *relA1*∆(*lacIZYA-argF*) U169 *deoR*(φ80d*lac*∆(*lacZ*)M15); used as a cloning host strain | (15) |
| S17-1::pZMR100 | S17-1 strain containing pZMR100 plasmid used to confer Km^r^; used as Km^r^ prey for *Bdellovibrio* | (16) |
| S17-1::pMal-p2_mCherry | S17-1 strain containing pMal-p2_mCherry plasmid; exports mCherry protein into *E. coli* periplasm, used for backlit microscopy | (17) |
| MG1655 | F- lambda- *ilvG*- *rfb*-50 *rph*-1 (K12 derivative) | (18) |
|  | *Bdellovibrio bacteriovorus* |  |
| HD100 | Type strain, genome sequenced | (19,20) |
| HID2 | HI derivative of wild-type HD100 | (11) |
| HID13 | HI derivative of wild-type HD100 | (11) |
| HID26 | HI derivative of wild-type HD100 | (11) |
| *dgcA* HI | ∆*dgcA* (∆*Bd0367*) | This study |
| *dgcB* HI | ∆*dgcB* (∆*Bd0742*) | This study |
| *dgcC:*Kmr HD | *dgcC*::Kmr (∆*Bd1434*::Kmr) | This study |
| *dgcC:*Kmr HI | HI derivative of *dgcC*::Kmr | This study |
| *dgcC* HD | ∆*dgcC* (∆*Bd1434*) | This study |
| *dgcD* HD | ∆*dgcD* (∆*Bd3766*) | This study |
| *dgcD* HI | HI derivative of ∆*dgcD* (∆*Bd3766*) | This study |
| *cdgA* HD | ∆*cdgA* (∆*Bd3125*) | This study |
| *dgcA*-mCherry HD | HD100 strain carrying integrated plasmid pK18:*dgcA*-mCherry at the *dgcA* (*Bd0367*) locus | This study |
| *dgcA*-mCherry HI | HI derivative of *dgcA*-mCherry | This study |
| *dgcB*-mCherry HD | HD100 strain carrying integrated plasmid pK18:*dgcB*-mCherry at the *dgcB* (*Bd0742*) locus | This study |
| *dgcB*-mCherry HI | HI derivative of *dgcB*-mCherry | This study |
| *dgcC*-mCherry HD | HD100 strain carrying integrated plasmid pK18:*dgcC*-mCherry at the *dgcC* (*Bd1434*) locus | This study |
| *dgcC*-mCherry HI | HI derivative of *dgcC*-mCherry | This study |
| *dgcD*-mCherry HD | HD100 strain carrying integrated plasmid pK18:*dgcD*-mCherry at the *dgcD* (*Bd3766*) locus | This study |
| *dgcD*-mCherry HI | HI derivative of *dgcD*-mCherry | This study |
| *cdgA*-mCherry HD | HD100 strain carrying integrated plasmid pK18:*cdgA*-mCherry at the *cdgA* (*Bd3125*) locus | This study |
| *cdgA*-mCherry HI | HI derivative of *cdgA*-mCherry | This study |
| *dgcA::* pK18:comp:*dgcA*  -mCherry HD | ∆*dgcA* (∆*Bd0367*) strain carrying integrated plasmid pK18:comp:*dgcA*-mCherry at the deletion site | This study |
| *dgcB::* pK18:comp:*dgcB* HD | ∆*dgcB* (∆*Bd0742*) strain carrying integrated plasmid pK18:comp:*dgcB* at the deletion site | This study |
| *dgcB::* pK18:comp:*dgcB*_GGAAF_ HI | ∆*dgcB* (∆*Bd0742*) strain carrying integrated plasmid pK18:comp:*dgcB*_GGAAF_ at the deletion site | This study |
| *dgcC*::pK18:comp:*dgcC* HD | ∆*dgcC* (∆*Bd1434*) strain carrying integrated plasmid pK18:comp:*dgcC* at the deletion site | This study |
| *dgcC*::pK18:comp:*dgcC*_GGAAF_ HD | ∆*dgcC* (∆*Bd1434*) strain carrying integrated plasmid pK18:comp:*dgcC*_GGAAF_ at the deletion site | This study |
| *dgcD*::pK18:comp:*dgcD* HD | ∆*dgcD* (∆*Bd3766*) strain carrying integrated plasmid pK18:comp:*dgcD* at the deletion site | This study |
| *cdgA*::pK18:comp:*cdgA* HD | ∆c*dgA* (∆*Bd3125*) strain carrying integrated plasmid pK18:comp:*cdgA* at the deletion site | This study |
|  | Plasmids |  |
| pK18*mobsacB* | Km^r^ suicide vector used for conjugation and recombination into *Bdellovibrio* genome | (21) |
| pSET151 | Suicide vector used for conjugation and recombination into *Bdellovibrio* genome | (22) |
| pMal-c2x | Vector for overexpression of maltose-binding protein (MBP) fusions | NEB Biolabs |
| pK18:∆*dgcA* | pK18*mobsacB* suicide plasmid containing 1kb of 5’- and 3’- flanking genomic DNA from around *dgcA* (*Bd0367*) | This study |
| pK18:∆*dgcB* | pK18*mobsacB* suicide plasmid containing 1kb of 5’- and 3’- flanking genomic DNA from around *dgcB* (*Bd0742*) | This study |
| pSET151:*dgcC*:Kmr | pSET151 suicide plasmid containing 1kb of 5’- and 3’- flanking genomic DNA from around *dgcC* (*Bd1434*), with Km^r^ cassette replacement of *dgcC* (*Bd1434*) ORF | This study |
| pK18:∆*dgcC* | pK18*mobsacB* suicide plasmid containing 1kb of 5’- and 3’- flanking genomic DNA from around *dgcC* (*Bd1434*) | This study |
| pK18:∆*dgcD* | pK18*mobsacB* suicide plasmid containing 1kb of 5’- and 3’- flanking genomic DNA from around *dgcD* (*Bd3766*) | This study |
| pK18:∆*cdgA* | pK18*mobsacB* suicide plasmid containing 1kb of 5’- and 3’- flanking genomic DNA from around c*dgA* (*Bd3125*) | This study |
| pK18:*dgcA*-mCherry | pK18*mobsacB* containing the ORF of *dgcA* (*Bd0367*) lacking the stop codon and fused in-frame with mCherry | This study |
| pK18:*dgcB*-mCherry | pK18*mobsacB* containing the ORF of *dgcB* (*Bd0742*) lacking the stop codon and fused in-frame with mCherry | This study |
| pK18:*dgcC*-mCherry | pK18*mobsacB* containing the ORF of *dgcC* (*Bd1434*) lacking the stop codon and fused in-frame with mCherry | This study |
| pK18:*dgcD*-mCherry | pK18*mobsacB* containing the ORF of *dgcD* (*Bd3766*) lacking the stop codon and fused in-frame with mCherry | This study |
| pK18:*cdgA*-mCherry | pK18*mobsacB* containing the ORF of c*dgA* (*Bd3125*) lacking the stop codon and fused in-frame with mCherry | This study |
| pK18:comp:*dgcA*-mCherry | pK18*mobsacB* containing the ORF of *dgcA* (*Bd0367*) lacking the stop codon and fused in-frame with mCherry with 1kb of 5’ flanking genomic DNA from *dgcA* (*Bd0367*) | This study |
| pK18:comp:*dgcB* | pK18*mobsacB* suicide plasmid containing the full ORF of *dgcB* along with 1kb of 5’- and 250bp of 3’- flanking genomic DNA from around *dgcB* (*Bd0742*) | This study |
| pK18:comp:*dgcB*_GGAAF_ | pK18*mobsacB* suicide plasmid containing the full ORF of *dgcB,* with mutated GGEEF to GGAAF, along with 1kb of 5’- and 250bp of 3’- flanking genomic DNA from around *dgcB* (*Bd0742*) | This study |
| pK18:comp:*dgcC* | pK18*mobsacB* suicide plasmid containing the full ORF of *dgcC* along with 1kb of 5’- and 250bp of 3’- flanking genomic DNA from around *dgcC* (*Bd1434*) | This study |
| pK18:comp:*dgcC*_GGAAF_ | pK18*mobsacB* suicide plasmid containing the full ORF of *dgcC,* with mutated GGEEF to GGAAF, along with 1kb of 5’- and 250bp of 3’- flanking genomic DNA from around *dgcC* (*Bd1434*) | This study |
| pK18:comp:*dgcD* | pK18*mobsacB* suicide plasmid containing the full ORF of *dgcD* along with 1kb of 5’- and 250bp of 3’- flanking genomic DNA from around *dgcD* (*Bd3766*) | This study |
| pK18:comp:*cdgA* | pK18*mobsacB* suicide plasmid containing the full ORF of *cdgA* along with 1kb of 5’- and 250bp of 3’- flanking genomic DNA from around *cdgA* (*Bd3125*) | This study |
| pMDgcA | pMal-c2x expressing the MBP-DgcA fusion | This study |
| pMDgcB | pMal-c2x expressing the MBP-DgcB fusion | This study |
| pMDgcC | pMal-c2x expressing the MBP-DgcC fusion | This study |
| pMGGDEF_DgcD_ | pMal-c2x expressing the MBP fusion to the GGDEF domain from DgcD | This study |
| pMCdgA | pMal-c2x expressing the MBP-CdgA fusion | This study |

**Table S2.** Oligonucleotides used in this study.

| **Gene and purpose** | **Primer** | | **RE^1^** |
| --- | --- | --- | --- |
| *dgcA* (*Bd0367*) KO construct | Forward | 5’- CCCAAGCTTATCGACCACTTCGTCGTTGG -3’ | *HindIII* |
|  | Internal Reverse | 5’- ACGTCGGTACCTTAGCCGGCCGCACTCATCGGTTAATTCCGGG -3’ | *KpnI* |
|  | Internal Forward | 5’- AACCGATGAGTGCGGCCGGCTAAGGTACCTTATTCGTT -3’ | *KpnI* |
|  | Reverse | 5’- CCCAAGCTTCATAATCCTTTACCACCGCC -3’ | *HindIII* |
| *dgcB* (*Bd0742*) KO construct | Forward | 5’- CGGGGTACCATTGCGGAAGTTTTCTGTGC -3’ | *KpnI* |
|  | Internal Reverse | 5’- TAGCCGCGGCCGCTTAAGCGACGATCGTCACCTGACAATCAAGCT -3’ | *NotI* |
|  | Internal Forward | 5’- GTCAGGTGACGATCGTCGCTTAAGCGGCCGCTTTTAGTTT -3’ | *NotI* |
|  | Reverse | 5’- CGGGGTACCAGTTCTTCAATTCAGCCTGC -3’ | *KpnI* |
| *dgcC* (*Bd1434*) KO construct (both Kmr and silent deletion) | Forward | 5’- CGGGGTACCACATGCACTTCCAGATTGCG -3’ | *KpnI* |
|  | Internal Reverse | 5’- ATCGGGATATCTCAGGCCACACGATACATTTAATTCTATTTAC -3’ | *EcoRV* |
|  | Internal Forward | 5’- ATTAAATGTATCGTGTGGCCTGAGATATCGGCACTGTC -3’ | *EcoRV* |
|  | Reverse | 5’- CGGGGTACCTAATTTCCTGCGGTGCCAGG -3’ | *KpnI* |
| *dgcD* (*Bd3766*) KO construct | Forward | 5’- CGGGGTACCAAGTTGTTGCTGAAGACCGC -3’ | *KpnI* |
|  | Internal Reverse | 5’- TAGCCGCGGCCGCTTACGACACCAAGGACAAAGCCGGGCTGGACT -3’ | *NotI* |
|  | Internal Forward | 5’- CGGCTTTGTCCTTGGTGTCGTAAGCGGCCGCACACACTAG -3’ | *NotI* |
|  | Reverse | 5’- CGGGGTACCTTTCCACGACAGGAGTATCC -3’ | *KpnI* |
| *cdgA* (*Bd3125*) KO construct | Forward | 5’- CGGGGTACCGATCACCAAAGACATTCAGG -3’ | *KpnI* |
|  | Internal Reverse | 5’- TAGCCGCGGCCGCCTATTCCGCTGTGTTCAATTAAATCCTCTTTC -3’ | *NotI* |
|  | Internal Forward | 5’- TTTAATTGAACACAGCGGAATAGGCGGCCGCAGACTAAGA -3’ | *NotI* |
|  | Reverse | 5’- CGGGGTACCTACAGATGCTTCCGACAAGG -3’ | *KpnI* |
| *dgcA* (*Bd0367*) mCherry tag construct | Forward | 5’- TTCCGAGCTCATGAGTAGGGCAGAAGTTACACTGG -3’ | *SacI* |
|  | Reverse | 5’- GGGGTACCGCCGGCCGCTTTACGACGC -3’ | *KpnI* |
| *dgcB* (*Bd0742*) mCherry tag construct | Forward | 5’- TTCCGAGCTCGTGACGGGGGGAAGTTTTATACTTTTCC -3’ | *SacI* |
|  | Reverse | 5’- GGGGTACCAGCGACGATGGTCGTACGG -3’ | *KpnI* |
| *dgcC* (*Bd1434*) mCherry tag construct | Forward | 5’- TTCCGAGCTCATGTATTTCTCAGTGTCTCAAGG -3’ | *SacI* |
|  | Reverse | 5’- GGGGTACCGGCCACACGTTTGATCTGGG -3’ | *KpnI* |
| *dgcD* (*Bd3766*) mCherry tag construct | Forward | 5’- TTCCGAGCTCTTGTCCGATGAATCTGGAAGTATGC -3’ | *SacI* |
|  | Reverse | 5’- GGGGTACCCGACACCAACCGCAAGCTTACG -3’ | *KpnI* |
| *cdgA* (*Bd3125*) mCherry tag construct | Forward | 5’- CGGGAATTCTTGAACATTCGCGATTACAG -3’ | *EcoRI* |
|  | Reverse | 5’- GGGGTACCTTCCGCTGTCACTTCAAATTCAGG -3’ | *KpnI* |
| *dgcA* (*Bd0367*) mCherry complement construct | Forward | 5’- TTCCGAGCTCTCGACCACTTCGTCGTTGGC -3’ | *SacI* |
|  | Reverse | 5’- TTCCGAGCTCATTCCGGGAATTATTTAGAC -3’ | *SacI* |
| *dgcB* (*Bd0742*) complement construct | Forward | 5’- TTAGGCCGGAATTCCGGAAGTTTTCTGTGCTCGC -3’ | *EcoRI* |
|  | Reverse | 5’- TTAGGCCGGAATTCTAAGCAGAGCTGACGGAAGG -3’ | *EcoRI* |
| *dgcB* (*Bd0742*) GGAAF mutation construct | Internal Reverse | 5’- CGGAAAGCAGCAGCACAAAGGCGGCACCACCGTAACGGGCAAAG -3’ | *-* |
|  | Internal Forward | 5’- CTTTGCCCGTTACGGTGGTGCCGCCTTTGTGCTGCTGCTTTCCG -3’ | *-* |
| *dgcC* (*Bd1434*) complement construct | Forward | 5’- TTAGGCGCGGATCCCACATGCACTTCCAGATTGC -3’ | *BamHI* |
|  | Reverse | 5’- TTAGGCGCGGATCCGCTAAAGCAAGCATGAAGCC -3’ | *BamHI* |
| *dgcC* (*Bd1434*) GGAAF mutation construct | Internal Reverse | 5’- CTGGCAAGATCACCGCGAAGGCGGCACCGCCCAGGCGGGCAATG -3’ | *-* |
|  | Internal Forward | 5’- CATTGCCCGCCTGGGCGGTGCCGCCTTCGCGGTGATCTTGCCAG -3’ | *-* |
| *dgcD* (*Bd3766*) complement construct | Forward | 5’- CGTGCTGCTCTAGAGCAACTTTCGAAGTTGTTGC -3’ | *BamHI* |
|  | Reverse | 5’- TTAGGCGCGGATCCTAATGCCAATCGCATTCTGG -3’ | *BamHI* |
| *cdgA* (*Bd3125*) complement construct | Forward | 5’- CGGGGTACCGATCACCAAAGACATTCAGG -3’ | *EcoRI* |
|  | Reverse | 5’- TTAGGCCGGAATTCCACACCCAAGTGCTTGATGC -3’ | *EcoRI* |
| *dgcA (Bd0367)* overexpression construct | Forward | 5’- CGCCGGATCCATGAGTAGGGCAGAAGTTACACTG -3’ | *BamHI* |
|  | Reverse | 5’- AAGGCTGCAGTTAGCCGGCCGCTTTACG -3’ | *PstI* |
| *dgcB(Bd0742)* overexpression construct | Forward | 5’- GACGGGATCCATGGCTCACAACGATGACAAC -3’ | *BamHI* |
|  | Reverse | 5’- ACGCGTCGACTTAAGCGACGATGGTCGTACG -3’ | *SalI* |
| *dgcC (Bd1434)* overexpression construct | Forward | 5’- AACAGGATCCATGGACTGGGGCTCGAACAG -3’ | *BamHI* |
|  | Reverse | 5’- CGCGTCTAGATCAGGCCACACGTTTGATCTG -3’ | *XbaI* |
| GGDEF_DgcD_ (GGDEF_Bd3766_) overexpression construct | Forward | 5’ - AGCCGGATCC ATCCGTCAGGTGCGCACCGCCCGT -3’ | *BamHI* |
|  | Reverse | 5’ - GAGCTCTAGATTACGACACCAACCGCAAGCTTAC -3’ | *XbaI* |
| *cdgA (Bd3125)* overexpression construct | Forward | 5’- GGCGTCTAGAGTGTATCTTTCACAAGCCGG -3’ | *XbaI* |
|  | Reverse | 5’- GCCCCTGCAGCTATTCCGCTGTCACTTCAAATTC -3’ | *PstI* |

^1^ Restriction enzyme sites introduced for cloning into plasmid vectors.

**Figure S1.** Predatory and axenic lifecycles of wild-type *Bdellovibrio* *bacteriovorus* HD100.


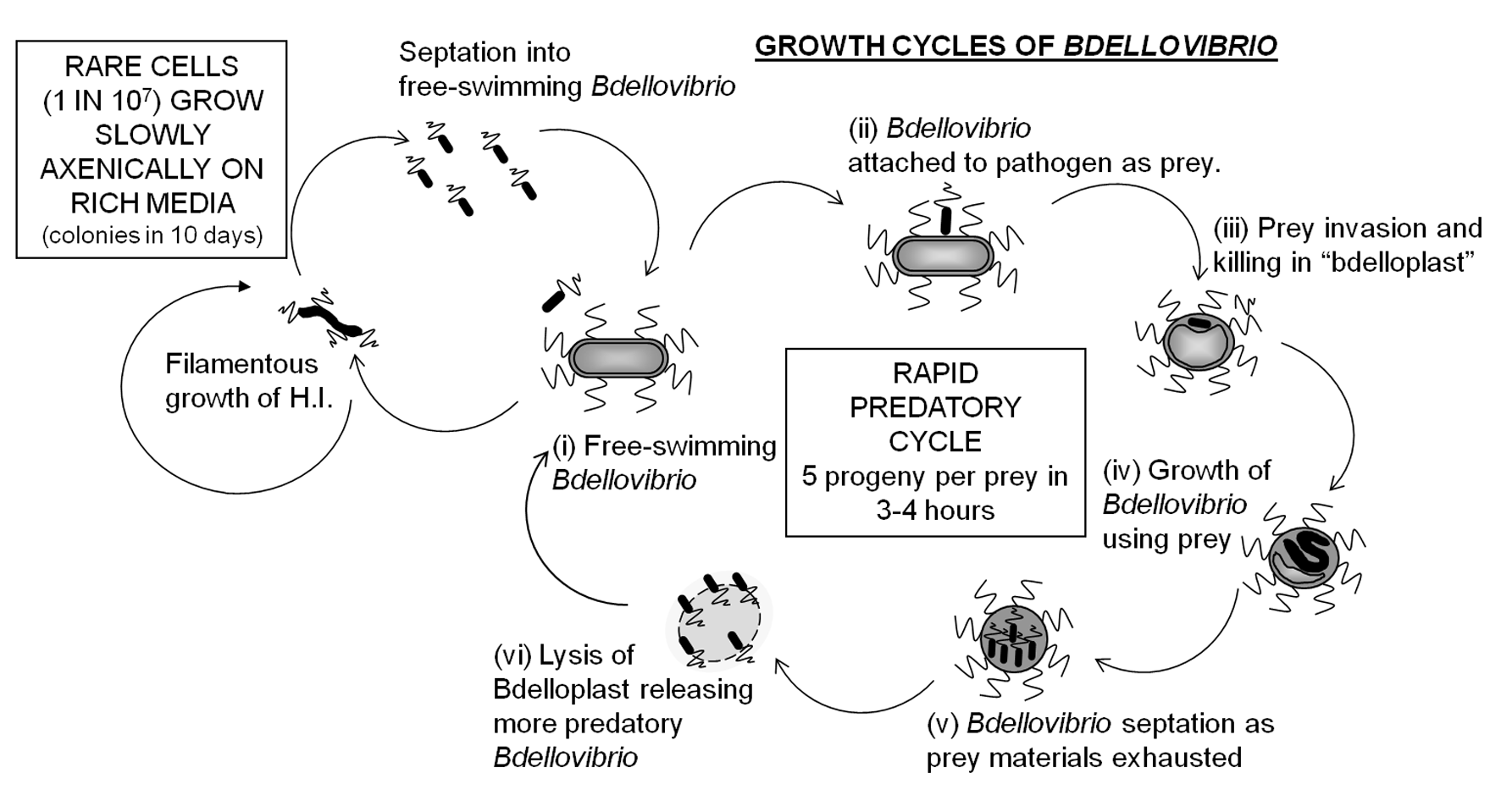


**Figure S2.** Pfam (23) Domain architectures and activities of 5 GGDEF domain proteins from *B.* *bacteriovorus*. REC, receiver domain of bacterial response regulators; FHA, forkhead domain; small yellow box, a signal sequence; small red box, a transmembrane domain; small green box, low complexity; crossed GGDEF domain, an enzymatically inactive domain; ND, not done.


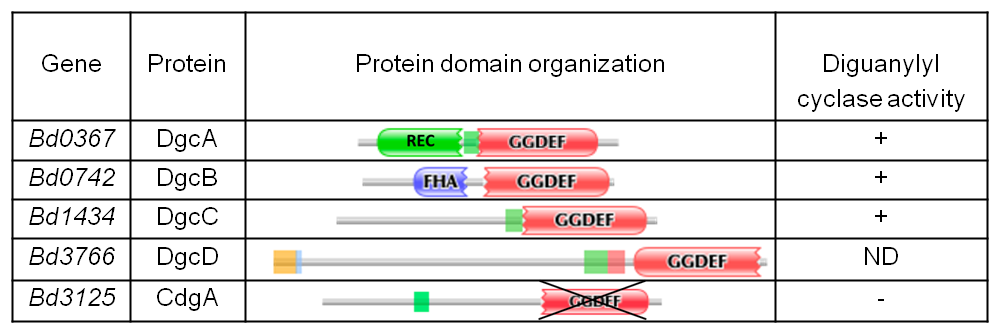


**Figure S3.** Effect on swimming in the semi-solid agar of the MBP-fusion proteins to the full-length DgcA, DgcB, DgcC, CdgA and the GGDEF domain of DgcD. Overnight cultures of *E.* *coli* MG1655 containing appropriate plasmids were concentrated, and 3 µl was inoculated onto soft agar plates containing 0.25% agar, 1% tryptone and 0.5% NaCl. Images were taken after 6 h at 37 °C.

**
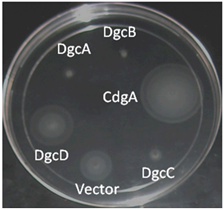
**

**Figure S4.** Complemented gliding phenotype produced by single recombination of full length *dgcA-mcherry* into the *∆dgcA* strain. Still snapshots taken from timelapse imaging shows complemented show that the complemented cell is able to glide across the agarose surface over a 2 hour interval (Compare to Fig 4 in main paper text) (Time shown in minutes).

**
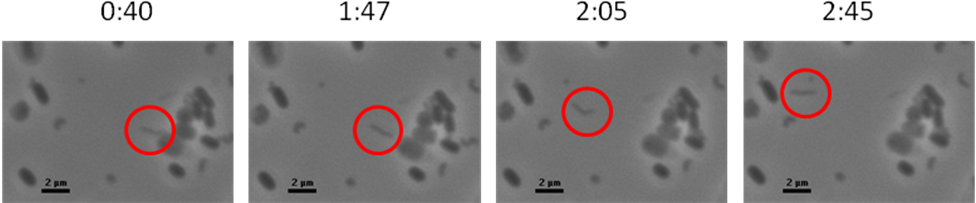
**

**Figure S5** Complemented (top) and original *∆cdgA* mutant HI cells regain the ability to attach to and enter prey cells in an hour or less, as for wild type HI *Bdellovibrio*. Still snapshots taken from timelapse microscopy show attachments of long thin *∆cdgA* wild type complemented *Bdellovibrio,* attaching to, rounding up and entering the larger darker prey *E. coli* cells 35 minutes in comparison to the 87 minutes taken for the uncomplemented *∆cdgA* strain. Time shown in minutes.

Compl. ∆*cdgA*  Start of invasion End of invasion


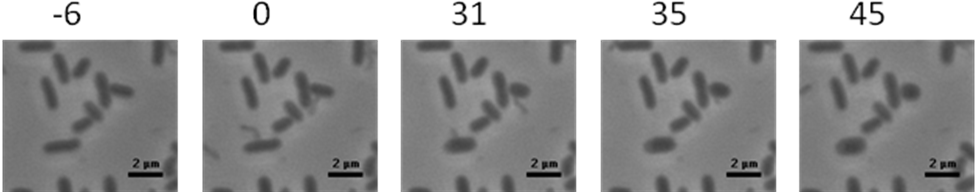


∆*cdgA* Start of invasion End of invasion


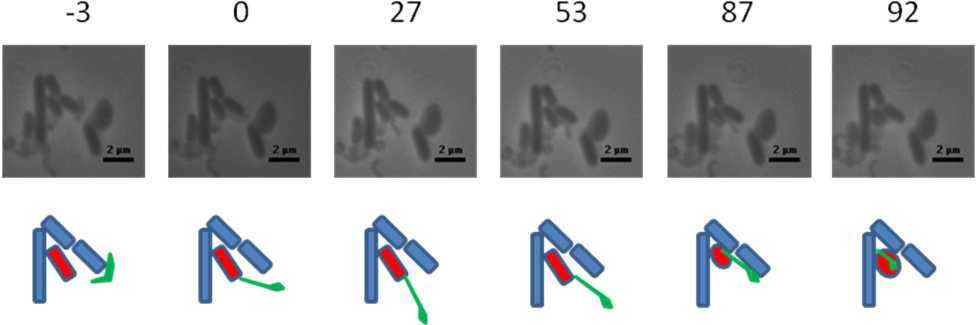


**Figure S6.** Timelapse imaging shows bipolar predation of two prey by a single long *Bdellovibrio* cell (in this case a wild type carrying mreB2-mTFP) the thin filamentous *Bdellovibrio* attaches at T=0 to a prey cell to the left of frame and by T=20 that prey is rounding up. At T=20 the second prey at the opposite *Bdellovibrio* pole is attached, and by T=40 that too is rounding up. From T=45-80 the *Bdellovibrio* pulls together the two prey invading and replicating within both and can be seen at T=255 growing within each prey bdelloplast. At T=273-280 septated *Bdellovibrio* progeny are released. This figure shows that in Fig 5E(vii) where bipolar foci of CdgA are seen on long *Bdellovibrio* cells, that these may be markers of functional bipolar “noses” for prey invasion.


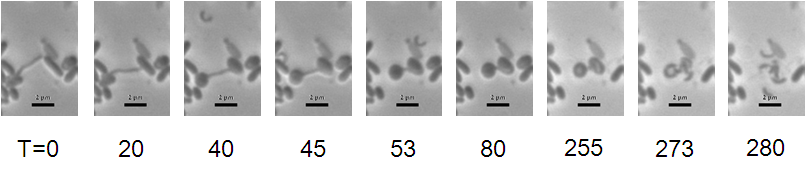

Supplement: Text S1 — SOM text. (DOC) [file ppat.1002493.s001.doc]
